# Inflammatory and Metabolic Biomarkers Associated with Chronic Subdural Hematoma Recurrence: A Single-Center Retrospective Cohort Study

**DOI:** 10.3390/clinpract16100193

**Published:** 2026-10-14

**Authors:** Bilal Ertuğrul, Harun Kömürcü

**Affiliations:** Department of Neurosurgery, Faculty of Medicine, Fırat University, 23100 Elazığ, Türkiye; hkomurcu@firat.edu.tr

**Keywords:** chronic subdural hematoma, recurrence, lactate, lactate/albumin ratio, biomarker, burr-hole evacuation, systemic inflammation, blood gas analysis, neurosurgery

## Abstract

**Background:** Chronic subdural hematoma (CSDH) recurs in 5–20% of patients despite surgical treatment, and readily accessible biomarkers associated with recurrence remain limited. This study evaluated perioperative hematological, biochemical, and metabolic biomarkers associated with CSDH recurrence, focusing on preoperative lactate and the lactate-to-albumin ratio (LAR). **Methods:** This retrospective single-center cohort study included 163 adults who underwent burr-hole evacuation for CSDH between January 2021 and December 2025. All patients underwent saline irrigation without placement of a postoperative subdural drain, in accordance with the institutional surgical protocol. Clinical, radiological, laboratory, and perioperative arterial blood gas data were analyzed. Recurrence was defined as symptomatic re-expansion requiring repeat surgery within six months. Receiver operating characteristic (ROC) analysis and multivariable logistic regression were used to assess discrimination and associations with recurrence. **Results:** Postoperative recurrence occurred in 22 patients (13.5%). Recurrence was associated with higher preoperative lactate, LAR, neutrophil-to-lymphocyte ratio, and systemic immune-inflammation index, as well as lower serum albumin. LAR showed moderate discrimination (AUC 0.758, 95% CI 0.652–0.864), while lactate had an AUC of 0.744. Increased LAR (OR 1.42 per 0.1-unit increase, 95% CI 1.15–1.76; *p* = 0.001), bilateral hematoma (OR 2.91, 95% CI 1.08–7.84; *p* = 0.035), and mixed-density hematoma (OR 2.64, 95% CI 1.01–6.89; *p* = 0.047) were independently associated with recurrence in the fitted model. The age × lactate interaction did not reach conventional statistical significance (*p* = 0.058) and was considered exploratory. **Conclusions:** Higher preoperative LAR was associated with CSDH recurrence and showed moderate discriminative performance in this cohort. These findings are exploratory and require independent validation before LAR or the combined model can be considered for clinical risk prediction.

## 1. Introduction

Chronic subdural hematoma (CSDH) is one of the most common neurosurgical conditions, particularly in the elderly, and its incidence continues to rise alongside the aging global population. Minor head trauma, cerebral atrophy, and the use of antithrombotic agents represent the most critical predisposing factors. Although burr-hole drainage remains the standard surgical approach for symptomatic patients, postoperative recurrence occurs in approximately 5–20% of cases despite successful surgery. Because recurrence necessitates repeat surgery, prolongs hospitalization, and increases morbidity, early identification of patients at high risk is of paramount clinical importance [1,2].

The pathophysiology of CSDH recurrence cannot be attributed solely to surgical factors. Persistent chronic inflammation, neoangiogenesis, increased vascular permeability, and recurrent microhemorrhages within the hematoma membrane are considered important mechanisms driving recurrence. Previous studies have identified advanced age, bilateral hematoma, marked midline shift, mixed-density hematoma, antithrombotic therapy, and inadequate brain re-expansion as factors associated with recurrence [3,4]. However, the role of readily available hematological, biochemical, and metabolic biomarkers in recurrence risk assessment remains incompletely defined.

In recent years, inflammatory biomarkers such as the neutrophil-to-lymphocyte ratio (NLR), platelet-to-lymphocyte ratio (PLR), and systemic immune-inflammation index (SII) have been investigated as prognostic indicators in neurosurgical disorders. In contrast, evidence specifically addressing serum lactate and the lactate-to-albumin ratio (LAR) in CSDH remains limited. LAR has been studied as a marker integrating metabolic stress with systemic inflammation or physiological reserve in other clinical settings [5]. The present study therefore focused primarily on the association between preoperative LAR and CSDH recurrence and compared its discriminative performance with lactate and routinely available inflammatory biomarkers within the same cohort.

Serum lactate may increase in response to tissue hypoperfusion, adrenergic stimulation, physiological stress, and altered cellular metabolism. Serum albumin, a negative acute-phase reactant, is influenced by systemic inflammation, nutritional status, chronic disease, and physiological reserve [6]. Thus, LAR may integrate several systemic processes [7,8]. Although inflammation, angiogenesis, and recurrent microhemorrhage are established features of CSDH pathophysiology, peripheral LAR should not be assumed to directly measure biological activity within the hematoma membrane. The possible relationship between systemic LAR and CSDH recurrence therefore remains a hypothesis requiring validation.

The primary analysis focused on the association between preoperative LAR and recurrence after surgical treatment of CSDH. Comparisons involving lactate, other routinely available inflammatory biomarkers, perioperative lactate measurements, and age-stratified findings were considered secondary and exploratory. By integrating metabolic and inflammatory markers with established clinical and radiological variables, the study sought to provide exploratory evidence regarding their potential role in recurrence risk stratification.

## 2. Materials and Methods

### 2.1. Study Design and Patient Selection

This study was designed as a single-center retrospective cohort study conducted at the Department of Neurosurgery, Fırat University Hospital. The clinical, radiological, and laboratory records of patients who underwent surgical treatment for chronic subdural hematoma (CSDH) between January 2021 and December 2025 were retrospectively reviewed. Patients aged 18 years or older with a cranial computed tomography (CT)-confirmed diagnosis of CSDH, who underwent burr-hole trephination, and who completed at least six months of clinical and radiological follow-up were included. Patients with acute subdural hematoma, subdural empyema, subdural collections secondary to malignancy, active systemic infection, or hematological malignancy were excluded. Patients with incomplete laboratory data, radiological imaging, or follow-up records were also excluded from the analysis.

### 2.2. Clinical Assessment

Patient demographics and clinical characteristics, including age, sex, hypertension, diabetes mellitus, coronary artery disease, chronic kidney disease, history of alcohol consumption, and history of head trauma, were recorded. The use of antiplatelet and anticoagulant medications was also documented. Antiplatelet and anticoagulant medications were discontinued preoperatively whenever clinically feasible. In patients requiring urgent surgical evacuation, surgery was not routinely delayed to allow a predefined drug-withdrawal period.

Clinical data obtained from the medical records included the Glasgow Coma Scale (GCS) score at admission and presenting symptoms, including headache, hemiparesis, altered consciousness, and gait disturbance.

### 2.3. Radiological Assessment

Preoperative cranial computed tomography (CT) examinations were independently evaluated by four neurosurgery specialists at our institution. Hematoma location (unilateral or bilateral), maximum hematoma thickness, midline shift, and hematoma density were assessed. Hematomas were classified as hypodense, isodense, or mixed density. A formal interobserver agreement analysis was not performed.

Midline shift was measured at the level of the septum pellucidum and recorded in millimeters. For statistical analysis, patients were categorized into two groups according to the degree of midline shift (≤10 mm and >10 mm). Maximum hematoma thickness was measured on the axial CT slice demonstrating the greatest hematoma width.

### 2.4. Surgical Procedure

All patients underwent hematoma evacuation under general anesthesia using one or two burr-hole trephinations. The decision to perform one or two burr holes was based on the extent of the hematoma. After evacuation, the hematoma cavity was irrigated with warm normal saline. Postoperative subdural drains were not routinely used, in accordance with the institutional surgical protocol during the study period.

The number of burr holes, the surgical technique used, and perioperative complications were recorded.

### 2.5. Laboratory Assessment

Preoperative complete blood count (CBC) analyses included white blood cell count, neutrophil count, lymphocyte count, monocyte count, eosinophil count, platelet count, hemoglobin, hematocrit, mean platelet volume (MPV), and red cell distribution width (RDW).

Preoperative biochemical analyses included serum glucose, urea, creatinine, sodium, potassium, total protein, albumin, and C-reactive protein (CRP). Coagulation parameters included prothrombin time (PT), international normalized ratio (INR), and activated partial thromboplastin time (aPTT). Fibrinogen and D-dimer levels were additionally analyzed in patients for whom these data were available.

Preoperative, intraoperative, and postoperative arterial blood gas parameters available in the clinical records were extracted, including pH, lactate, bicarbonate (HCO_3_^−^), base excess, partial pressure of carbon dioxide (pCO_2_), partial pressure of oxygen (pO_2_), glucose, hemoglobin, sodium, potassium, and ionized calcium. Arterial blood gas analyses were performed using an ABL800 FLEX blood gas analyzer (Radiometer Medical ApS, Brønshøj to Copenhagen, Denmark). Preoperative lactate concentrations were obtained from arterial blood gas samples collected at initial hospital admission, before surgery and prior to induction of general anesthesia. The exact timing of intraoperative and postoperative arterial blood gas sampling was not completely standardized across all patients.

Postoperative CBC analyses included white blood cell count, neutrophil count, lymphocyte count, monocyte count, platelet count, hemoglobin, and hematocrit. Absolute changes between preoperative and postoperative measurements were also calculated.

LLAR = Preoperative lactate (mmol/L)/Preoperative serum albumin (g/dL)

To evaluate systemic inflammation, the following hematological indices were calculated:

Neutrophil-to-Lymphocyte Ratio (NLR) = Neutrophil count/Lymphocyte count

Platelet-to-Lymphocyte Ratio (PLR) = Platelet count/Lymphocyte count

Systemic Immune-Inflammation Index (SII) = (Platelet count × Neutrophil count)/Lymphocyte count.

### 2.6. Definition of Recurrence

Recurrence was defined as symptomatic re-expansion of the hematoma confirmed by follow-up cranial computed tomography (CT) within six months after the initial surgical procedure, accompanied by clinical deterioration and requiring repeat surgical intervention. Routine postoperative CT examinations were performed in the early postoperative period and at the 1-month follow-up.

Patients with residual or asymptomatic hematomas detected on follow-up imaging who did not require reoperation were not considered to have recurrence.

### 2.7. Statistical Analysis

Statistical analyses were performed using IBM SPSS Statistics for Windows, Version 26.0 (IBM Corp., Armonk, NY, USA).

The normality of continuous variables was assessed using the Shapiro–Wilk test. Normally distributed variables are presented as mean ± standard deviation (SD), whereas non-normally distributed variables are presented as median (interquartile range [IQR]). Categorical variables are expressed as frequency (*n*) and percentage (%).

Comparisons between the recurrence and non-recurrence groups were performed using the independent-samples *t*-test for normally distributed continuous variables and the Mann–Whitney U test for non-normally distributed variables. Categorical variables were compared using the Pearson chi-square test or Fisher’s exact test, as appropriate.

For the exploratory age-based subgroup analysis, patients were stratified into two groups: <65 years and ≥65 years. The age threshold of 65 years was used for exploratory stratification rather than as a biologically validated cutoff for CSDH recurrence. Preoperative lactate levels and LAR were compared between these age groups, and the age × lactate interaction was examined using logistic regression. Because the interaction did not reach conventional statistical significance, age-stratified findings were interpreted as exploratory and hypothesis-generating.

The association between preoperative LAR and recurrence constituted the primary analysis, whereas analyses of other biomarkers, perioperative lactate measurements, and age-stratified findings were considered secondary and exploratory. Variables considered for the multivariable logistic regression model were guided by univariable findings (*p* < 0.10), clinical relevance, correlations among candidate variables, and the limited number of recurrence events. The final model included LAR, bilateral hematoma, and mixed-density hematoma. With 22 recurrence events and three covariates, the events-per-variable (EPV) ratio was approximately 7.3. The model was intentionally kept parsimonious; nevertheless, the relatively low EPV may increase the risk of model instability and overfitting.

The discriminative performance of preoperative lactate and LAR was evaluated using receiver operating characteristic (ROC) curve analysis. The area under the curve (AUC), 95% confidence interval (CI), sensitivity, specificity, and the optimal cutoff based on the Youden index were calculated. The cutoff values were derived and evaluated within the same cohort and were therefore considered exploratory rather than clinically validated thresholds. No formal comparison of correlated AUCs using the DeLong test was performed; consequently, superiority of LAR over lactate was not inferred.

All statistical tests were two-tailed, and a *p*-value < 0.05 was considered statistically significant. No formal adjustment for multiple comparisons was applied because the secondary biomarker analyses were exploratory; therefore, these findings were interpreted as hypothesis-generating.

## 3. Results

### 3.1. Patient Characteristics

**Figure 1:**
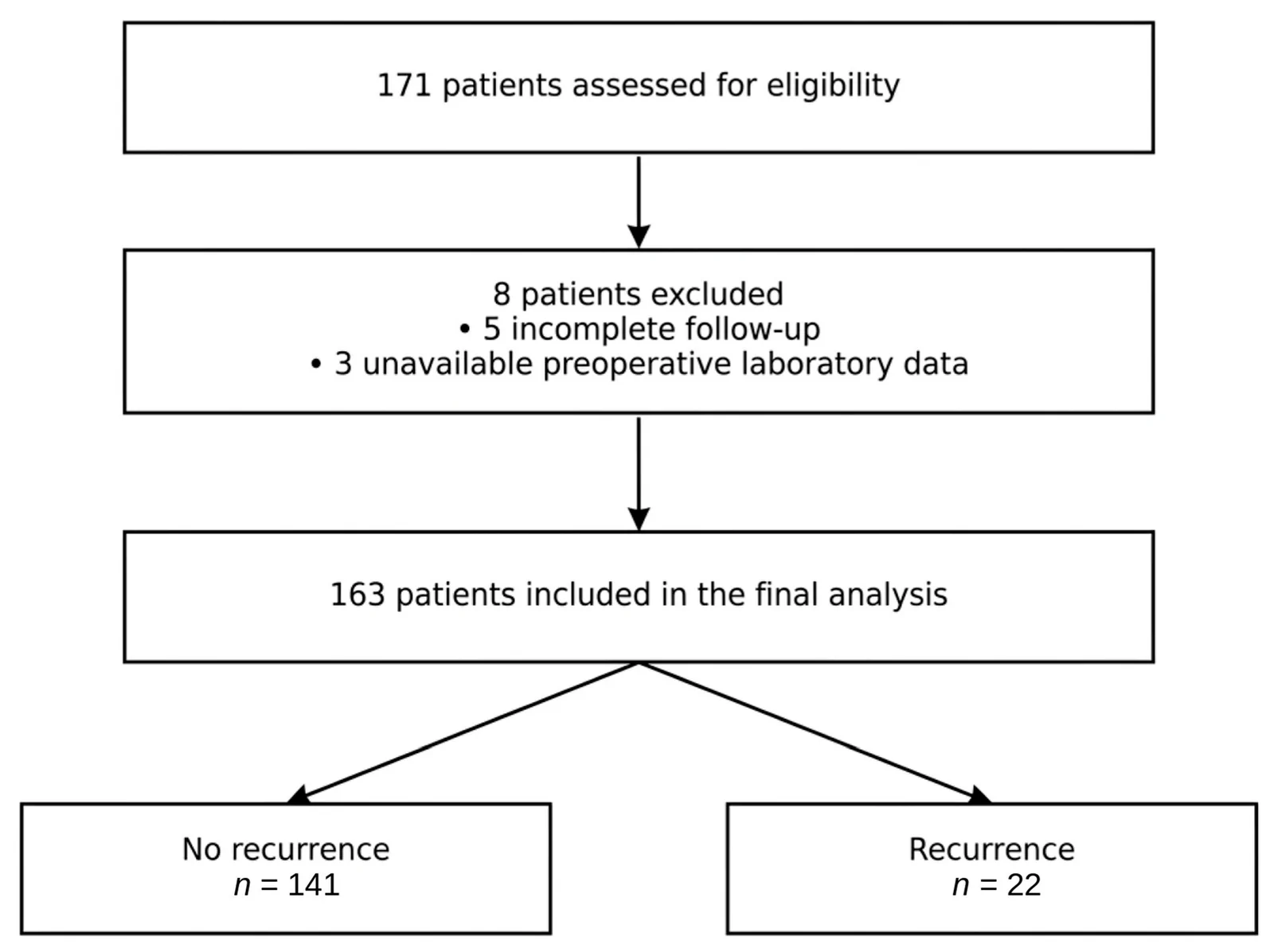
Patient selection flow diagram.

Between January 2021 and December 2025, 171 patients who underwent surgical treatment for CSDH were assessed for eligibility. Five patients with incomplete follow-up data and three patients without available preoperative laboratory data were excluded, leaving 163 patients in the final analysis (Figure 1).

The mean age of the study population was 63.2 ± 13.1 years (range, 44–89 years). Of the patients, 111 (68.1%) were male and 52 (31.9%) were female. Hypertension was present in 87 patients (53.4%), diabetes mellitus in 44 (27.0%), coronary artery disease in 38 (23.3%), and a history of regular alcohol consumption in 13 (8.0%).

Overall, 52 patients (31.9%) were receiving antiplatelet therapy and 18 (11.0%) were receiving anticoagulant therapy. A total of 63 patients (38.7%) were receiving any form of antithrombotic treatment.

Radiological evaluation demonstrated unilateral CSDH in 133 patients (81.6%) and bilateral CSDH in 30 patients (18.4%). The mean maximum hematoma thickness was 20.6 ± 5.9 mm, and the mean midline shift was 8.7 ± 5.1 mm. A midline shift of >10 mm was observed in 70 patients (42.9%).

Based on preoperative cranial computed tomography (CT), hematomas were classified as hypodense in 109 patients (66.9%), isodense in 10 (6.1%), and mixed density in 44 (27.0%).

### 3.2. Recurrence Outcomes

Postoperative recurrence requiring repeat surgery occurred in 22 patients (13.5%). The median time to recurrence was 61 days (interquartile range [IQR], 35–98 days).

The mean age was 66.8 ± 11.9 years in the recurrence group and 62.6 ± 13.2 years in the non-recurrence group, with no significant difference between the groups (*p* = 0.147).

The proportion of male patients was 81.8% (18/22) in the recurrence group and 66.0% (93/141) in the non-recurrence group (*p* = 0.142).

Diabetes mellitus was present in 40.9% (9/22) of patients with recurrence and 24.8% (35/141) of those without recurrence (*p* = 0.112).

Antithrombotic therapy was used in 50.0% (11/22) of patients in the recurrence group and 36.9% (52/141) in the non-recurrence group (*p* = 0.240).

Bilateral hematoma was identified in 36.4% (8/22) of patients with recurrence compared with 15.6% (22/141) of those without recurrence, representing a significantly higher prevalence in the recurrence group (*p* = 0.021).

A preoperative midline shift of >10 mm was observed in 68.2% (15/22) of patients with recurrence and 39.0% (55/141) of those without recurrence (*p* = 0.011).

Mixed-density hematoma was present in 14 patients (63.6%) in the recurrence group and 30 patients (21.3%) in the non-recurrence group. The prevalence of mixed-density hematoma was significantly higher among patients with recurrence (*p* < 0.001) (Table 1).

### 3.3. Hematological Findings

The preoperative white blood cell count was 9.31 ± 2.42 × 10^3^/µL in the recurrence group and 8.58 ± 2.19 × 10^3^/µL in the non-recurrence group (*p* = 0.153).

The preoperative neutrophil count was significantly higher in the recurrence group (6.78 ± 2.07 × 10^3^/µL) than in the non-recurrence group (5.83 ± 1.89 × 10^3^/µL, *p* = 0.031). The corresponding lymphocyte counts were 1.68 ± 0.61 × 10^3^/µL and 1.89 ± 0.65 × 10^3^/µL, respectively (*p* = 0.149).

The neutrophil-to-lymphocyte ratio (NLR) was significantly higher in the recurrence group (4.41 ± 2.08) than in the non-recurrence group (3.37 ± 1.64, *p* = 0.009).

No significant differences were observed in preoperative platelet count (247 ± 69 vs. 258 ± 72 × 10^3^/µL, *p* = 0.501) or platelet-to-lymphocyte ratio (PLR) (161 ± 72 vs. 145 ± 61, *p* = 0.259).

The systemic immune-inflammation index (SII) was significantly higher in the recurrence group (1026 ± 511) than in the non-recurrence group (754 ± 398, *p* = 0.005).

Preoperative hemoglobin levels were 12.8 ± 1.7 g/dL in the recurrence group and 13.2 ± 1.6 g/dL in the non-recurrence group (*p* = 0.274). The corresponding red cell distribution width (RDW) values were 14.7 ± 1.8% and 14.1 ± 1.6%, respectively (*p* = 0.106).

The postoperative neutrophil-to-lymphocyte ratio tended to be higher in the recurrence group (5.83 ± 2.71) than in the non-recurrence group (4.79 ± 2.20), although the difference did not reach statistical significance (*p* = 0.052).

The mean reduction in platelet count between the preoperative and postoperative measurements was significantly greater in the recurrence group (12.4 ± 9.8%) than in the non-recurrence group (7.1 ± 8.6%, *p* = 0.012).

### 3.4. Biochemical and Metabolic Findings

The preoperative serum albumin level was significantly lower in the recurrence group than in the non-recurrence group (3.78 ± 0.42 vs. 4.05 ± 0.39 g/dL, *p* = 0.004).

The median C-reactive protein (CRP) level was 8.9 mg/L in the recurrence group and 5.7 mg/L in the non-recurrence group, although the difference did not reach statistical significance (*p* = 0.071).

Preoperative lactate levels were significantly higher in the recurrence group than in the non-recurrence group (2.00 ± 0.71 vs. 1.43 ± 0.48 mmol/L, *p* < 0.001).

Similarly, intraoperative lactate levels were higher in the recurrence group (2.31 ± 0.82 vs. 1.77 ± 0.62 mmol/L, *p* = 0.001). Postoperative lactate levels also remained significantly higher in the recurrence group (1.83 ± 0.66 vs. 1.51 ± 0.53 mmol/L, *p* = 0.012).

There was no significant difference in preoperative pH between the recurrence and non-recurrence groups (7.40 ± 0.04 vs. 7.41 ± 0.04, *p* = 0.221). However, intraoperative pH was significantly lower in the recurrence group (7.36 ± 0.05 vs. 7.38 ± 0.04, *p* = 0.041).

Preoperative base excess was −1.9 ± 2.6 mmol/L in the recurrence group and −1.1 ± 2.3 mmol/L in the non-recurrence group (*p* = 0.137). Postoperative base excess values were −2.3 ± 2.8 mmol/L and −1.4 ± 2.4 mmol/L, respectively (*p* = 0.114).

The lactate-to-albumin ratio (LAR) was significantly higher in the recurrence group than in the non-recurrence group (0.54 ± 0.22 vs. 0.36 ± 0.14, *p* < 0.001) (Table 2).

### 3.5. Age-Based Subgroup Analysis of Lactate

Patients were stratified into two age groups: <65 years (*n* = 76) and ≥65 years (*n* = 87).

Recurrence occurred in 9 patients (11.8%) in the younger group and 13 patients (14.9%) in the older group (*p* = 0.560).

Among patients younger than 65 years, the preoperative lactate level was significantly higher in those with recurrence than in those without recurrence (2.42 ± 0.62 vs. 1.39 ± 0.44 mmol/L, *p* < 0.001).

In patients aged ≥65 years, preoperative lactate levels were 1.71 ± 0.59 mmol/L in the recurrence group and 1.46 ± 0.51 mmol/L in the non-recurrence group (*p* = 0.119).

Among patients with recurrence, those younger than 65 years had significantly higher preoperative lactate levels than those aged ≥65 years (2.42 ± 0.62 vs. 1.71 ± 0.59 mmol/L, *p* = 0.018).

Similarly, the LAR was significantly higher in younger patients with recurrence than in older patients with recurrence (0.64 ± 0.19 vs. 0.47 ± 0.20, *p* = 0.049).

The age × lactate interaction term in logistic regression was OR 0.96 (95% CI, 0.92–1.00; *p* = 0.058). Because the interaction did not reach conventional statistical significance, the observed subgroup differences were considered exploratory and hypothesis-generating rather than evidence of a confirmed age-dependent effect (Table 3).

### 3.6. ROC Curve Analysis

The area under the receiver operating characteristic (ROC) curve (AUC) for preoperative lactate in predicting recurrence was 0.744 (95% CI, 0.632–0.856; *p* < 0.001). A cutoff value of 1.75 mmol/L yielded a sensitivity of 68.2% and a specificity of 72.3%.

The AUC for the lactate-to-albumin ratio was 0.758 (95% CI, 0.652–0.864; *p* < 0.001). A cutoff value of 0.44 provided a sensitivity of 72.7% and a specificity of 70.2%.

The AUCs for the neutrophil-to-lymphocyte ratio (NLR) and systemic immune-inflammation index (SII) were 0.669 (95% CI, 0.548–0.790; *p* = 0.011) and 0.687 (95% CI, 0.573–0.801; *p* = 0.005), respectively.

Among the evaluated biomarkers, LAR had the numerically highest AUC (0.758); however, its AUC was close to that of preoperative lactate (0.744), and no formal comparison of the correlated ROC curves was performed. Therefore, these results should not be interpreted as demonstrating statistical superiority of LAR over lactate.

### 3.7. Multivariable Analysis

Based on the results of the univariable analysis, the correlations among significant variables, and the limited number of recurrence events, the lactate-to-albumin ratio (LAR), bilateral hematoma, and mixed-density hematoma were included in the multivariable logistic regression model.

Each 0.1-unit increase in LAR was associated with a 1.42-fold increase in the odds of recurrence (OR, 1.42; 95% CI, 1.15–1.76; *p* = 0.001).

The presence of bilateral hematoma was independently associated with a 2.91-fold increased risk of recurrence (OR, 2.91; 95% CI, 1.08–7.84; *p* = 0.035).

Mixed-density hematoma remained independently associated with recurrence after adjustment for the other clinical and radiological variables included in the model (OR, 2.64; 95% CI, 1.01–6.89; *p* = 0.047).

The final multivariable model had an apparent AUC of 0.811 (95% CI, 0.720–0.902; *p* < 0.001). Because this estimate was obtained in the same cohort used to fit the model and no internal or external validation or calibration analysis was performed, the AUC should be interpreted as an unvalidated estimate of discrimination rather than evidence of established clinical predictive performance (Table 4).

## 4. Discussion

In this retrospective cohort of patients with CSDH treated by burr-hole evacuation, recurrence requiring reoperation occurred in 13.5% of cases. The principal finding was the association between higher preoperative LAR and recurrence, while bilateral and mixed-density hematomas also remained associated with recurrence after multivariable adjustment. In addition, patients with recurrence had higher preoperative lactate, NLR, and SII levels and lower serum albumin levels.

LAR combines two physiologically distinct parameters. Lactate may increase in response to metabolic stress, altered tissue perfusion, or systemic stress [9], whereas albumin is influenced by nutritional status, inflammation, and physiological reserve [10,11]. Their combination may therefore capture aspects of systemic vulnerability that are not reflected by either parameter alone. Nevertheless, peripheral LAR cannot be regarded as a direct measure of CSDH membrane activity. Although inflammation, angiogenesis, increased vascular permeability, and recurrent microhemorrhage are established features of CSDH pathophysiology [12,13,14], the biological pathway linking peripheral LAR to hematoma recurrence remains uncertain.

The accompanying differences in NLR and SII further suggest a possible relationship between systemic inflammatory status and recurrence. Previous studies have also investigated systemic inflammatory indices in relation to CSDH recurrence [15]. These indices, however, were evaluated as secondary biomarkers in the present study, and the findings do not establish an independent role for either marker. Similarly, lower albumin and higher lactate may reflect broader systemic conditions rather than mechanisms specific to CSDH.

Radiological characteristics also appeared relevant. Bilateral and mixed-density hematomas were associated with recurrence after multivariable adjustment. Mixed density may reflect different stages of blood degradation and repeated hemorrhagic activity within the hematoma [16,17], whereas bilateral disease may represent a more extensive pathological process. These observations provide a radiological context for the laboratory findings but should not be interpreted as establishing causality.

LAR showed moderate discrimination for recurrence (AUC = 0.758), while the AUC for lactate was 0.744. Although the numerical value was slightly higher for LAR, no formal comparison between the two correlated ROC curves was performed. The LAR cutoff of 0.44 and the apparent AUC of 0.811 for the combined model were both obtained from the same cohort in which they were developed. The exploratory age × lactate interaction did not reach conventional statistical significance (*p* = 0.058). Accordingly, these estimates and subgroup findings should be regarded as preliminary and require validation in independent populations before clinical application.

## 5. Limitations

This study has several limitations. First, its retrospective, single-center design and the relatively small number of recurrence events may limit generalizability and increase the risk of residual confounding. Although the multivariable model was intentionally kept parsimonious, the 22 recurrence events resulted in an events-per-variable ratio of approximately 7.3, which may affect model stability.

Second, several clinical and perioperative factors may influence circulating lactate and albumin levels. Although preoperative lactate was obtained at initial hospital admission before surgery and induction of anesthesia, unmeasured differences in systemic condition and comorbidities may have influenced these biomarkers. In particular, the between-group difference in diabetes mellitus did not reach statistical significance, but residual confounding related to metabolic comorbidity cannot be excluded.

Third, the absence of routine postoperative subdural drainage reflects the institutional surgical protocol during the study period and may limit the applicability of the recurrence rates and associated factors to centers using routine drainage.

Finally, the ROC-derived cutoff values and the combined model were developed and evaluated in the same cohort. No penalized regression, bootstrap internal validation, external validation, or formal calibration assessment was performed. Therefore, the reported discrimination estimates may be optimistic and should be validated in larger independent cohorts.

## 6. Conclusions

Higher preoperative LAR was independently associated with recurrence after burr-hole evacuation of CSDH and showed moderate discriminatory ability. Bilateral and mixed-density hematomas were also independently associated with recurrence. However, these findings are based on a single-center retrospective cohort with a limited number of recurrence events. The proposed LAR cutoff and the combined model require validation in independent cohorts before they can be considered for clinical prediction or decision-making.

## Figures and Tables

**Table 1:** Baseline Demographic, Clinical, and Radiological Characteristics of the Study Population According to Recurrence Status.

Variable	No Recurrence (*n* = 141)	Recurrence (*n* = 22)	*p*-Value
Age (years), mean ± SD	62.6 ± 13.2	66.8 ± 11.9	0.147
Male sex, *n* (%)	93 (66.0)	18 (81.8)	0.142
Diabetes mellitus, *n* (%)	35 (24.8)	9 (40.9)	0.112
Antithrombotic therapy, *n* (%)	52 (36.9)	11 (50.0)	0.240
Hematoma laterality, *n* (%)			
Unilateral	119 (84.4)	14 (63.6)	—
Bilateral	22 (15.6)	8 (36.4)	0.021
Midline shift > 10 mm, *n* (%)	55 (39.0)	15 (68.2)	0.011
Mixed-density hematoma, *n* (%)	30 (21.3)	14 (63.6)	<0.001

**Table 2:** Comparison of Perioperative Hematological, Biochemical, and Metabolic Parameters Between Patients With and Without Recurrence.

Variable	No Recurrence (*n* = 141)	Recurrence (*n* = 22)	*p*-Value
H ematological parameters			
Neutrophils (×10^3^/µL)	5.83 ± 1.89	6.78 ± 2.07	0.031
Lymphocytes (×10^3^/µL)	1.89 ± 0.65	1.68 ± 0.61	0.149
Neutrophil-to-lymphocyte ratio	3.37 ± 1.64	4.41 ± 2.08	0.009
Platelet-to-lymphocyte ratio	145 ± 61	161 ± 72	0.259
Systemic immune-inflammation index	754 ± 398	1026 ± 511	0.005
Platelet decrease (%)	7.1 ± 8.6	12.4 ± 9.8	0.012
Biochemical parameters			
Albumin (g/dL)	4.05 ± 0.39	3.78 ± 0.42	0.004
C-reactive protein (mg/L)	5.7	8.9	0.071
Metabolic parameters			
Preoperative lactate (mmol/L)	1.43 ± 0.48	2.00 ± 0.71	<0.001
Intraoperative lactate (mmol/L)	1.77 ± 0.62	2.31 ± 0.82	0.001
Postoperative lactate (mmol/L)	1.51 ± 0.53	1.83 ± 0.66	0.012
Lactate-to-albumin ratio	0.36 ± 0.14	0.54 ± 0.22	<0.001

**Table 3:** Age-Based Subgroup Analysis of Preoperative Lactate and Lactate-to-Albumin Ratio.

Variable	<65 Years (*n* = 76)	≥65 Years (*n* = 87)	*p*-Value
Recurrence, *n* (%)	9 (11.8)	13 (14.9)	0.560
P reoperative lactate (mmol/L)			
Recurrence group	2.42 ± 0.62	1.71 ± 0.59	0.018 *
Non-recurrence group	1.39 ± 0.44	1.46 ± 0.51	—
Within-group *p*-value †	<0.001	0.119	—
Lactate-to-albumin ratio			
Recurrence group	0.64 ± 0.19	0.47 ± 0.20	0.049 *

* *p*-value for the comparison of the recurrence group between age strata (<65 vs. ≥65 years). † *p*-value for the comparison between the recurrence and non-recurrence groups within each age stratum. Age × lactate interaction term in the logistic regression model: odds ratio (OR) 0.96, 95% CI 0.92–1.00, *p* = 0.058.

**Table 4:** Receiver Operating Characteristic Curve and Multivariable Logistic Regression Analyses for Predicting Chronic Subdural Hematoma Recurrence.

**(a) Receiver Operating Characteristic Analysis**					
**Biomarker**	**Parameter/OR**	**95% CI**	**Cut-Off/*p*-Value**	**Sens. (%)**	**Spec. (%)**
ROC Curve Analysis	AUC				
Preoperative lactate	0.744	0.632–0.856	1.75	68.2	72.3
Lactate-to-albumin ratio	0.758	0.652–0.864	0.44	72.7	70.2
Neutrophil-to-lymphocyte ratio	0.669	0.548–0.790	—	—	—
Systemic immune-inflammation index	0.687	0.573–0.801	—	—	—
**(b) Multivariable Logistic Regression Analysis**					
**Variable**	**Odds Ratio**	**95% CI**		***p*-Value**	
Lactate-to-albumin ratio (per 0.1-unit increase)	1.42	1.15–1.76		0.001	
Bilateral hematoma	2.91	1.08–7.84		0.035	
Mixed-density hematoma	2.64	1.01–6.89		0.047	

Abbreviations: ROC, receiver operating characteristic; AUC, area under the curve; OR, odds ratio; CI, confidence interval; Sens., sensitivity; Spec., specificity.

## Data Availability

The datasets generated and/or analyzed during the current study are available from the corresponding author on reasonable request.

## Abbreviations

The following abbreviations are used in this manuscript:

aPTT	Activated Partial Thromboplastin Time
AUC	Area Under the Curve
CBC	Complete Blood Count
CI	Confidence Interval
CRP	C-Reactive Protein
CSDH	Chronic Subdural Hematoma
CT	Computed Tomography
GCS	Glasgow Coma Scale
INR	International Normalized Ratio
IQR	Interquartile Range
LAR	Lactate-to-Albumin Ratio
MPV	Mean Platelet Volume
NLR	Neutrophil-to-Lymphocyte Ratio
OR	Odds Ratio
PLR	Platelet-to-Lymphocyte Ratio
PT	Prothrombin Time
RDW	Red Cell Distribution Width
ROC	Receiver Operating Characteristic
SD	Standard Deviation
SII	Systemic Immune-Inflammation Index
WBC	White Blood Cell

## References

[1] Chen H., Colasurdo M., Bodanapally U.K., Malhotra A., Gandhi D. (2025). Chronic subdural hematoma: A review of current knowledge, treatment modalities, and clinical trials of middle meningeal artery embolization. Neurol. Clin. Pract..

[2] Zhong D., Cheng H., Xian Z., Ren Y., Li H., Ou X., Liu P. (2024). Advances in pathogenic mechanisms, diagnostic methods, surgical and non-surgical treatment, and potential recurrence factors of chronic subdural hematoma: A review. Clin. Neurol. Neurosurg..

[3] SeyedAlinaghi S., Mehraeen E., Farahani Rad F., Fayedeh F., Amiri Fard I., Siami H., Emamgholizadeh Baboli E., Molla A., Fakharian E., Tafakhori A. (2025). Risk factors of chronic subdural hematoma recurrence: A systematic review. Med. J. Islam. Repub. Iran.

[4] Bhatt P.B., Luo J., Mensah E.O., de C Caldas G., Marwah S., Taussky P., Ogilvy C.S. (2026). Molecular and pathophysiological mechanisms underlying chronic subdural hematoma recurrence: A comprehensive review. Neuromol. Med..

[5] Georgountzos G., Gkalonakis I., Anastasopoulos L., Stranjalis G., Kalamatianos T. (2023). Biofluid biomarkers in the prognosis of chronic subdural hematoma: A systematic scoping review. Diagnostics.

[6] Gharipour A., Razavi R., Gharipour M., Mukasa D. (2020). Lactate/albumin ratio: An early prognostic marker in critically ill patients. Am. J. Emerg. Med..

[7] Zhao X., Peng Q., Li W., Hu D., Guan Y., Wang J. (2024). Elevated lactate/albumin ratio is associated with poor prognosis in sepsis patients: A systematic review and meta-analysis. J. Med. Biochem..

[8] Suzuki Y., Aoki Y., Shimizu M., Nakajima M., Imai R., Okada Y., Mimuro S., Nakajima Y. (2024). Predictive accuracy of lactate albumin ratio for mortality in intensive care units: A nationwide cohort study. BMJ Open.

[9] Andersen L.W., Mackenhauer J., Roberts J.C., Berg K.M., Cocchi M.N., Donnino M.W. (2013). Etiology and therapeutic approach to elevated lactate levels. Mayo Clin. Proc..

[10] Don B.R., Kaysen G. (2004). Serum albumin: Relationship to inflammation and nutrition. Semin. Dial..

[11] Vincent J.L., Dubois M.J., Navickis R.J., Wilkes M.M. (2003). Hypoalbuminemia in acute illness: Is there a rationale for intervention? A meta-analysis of cohort studies and controlled trials. Ann. Surg..

[12] Kuczyński N., Pilewski D., Zomkowska E., Pulka W., Sowa M. (2026). Chronic subdural hematoma: Pathophysiology, diagnosis, and the emerging role of middle meningeal artery embolization. J. Clin. Med..

[13] Shono T., Inamura T., Morioka T., Matsumoto K., Suzuki S.O., Ikezaki K., Iwaki T., Fukui M. (2001). Vascular endothelial growth factor in chronic subdural haematomas. J. Clin. Neurosci..

[14] Sakthiyendran N.A., Gonzalez-Salido J., Perkins T., Enriquez-Marulanda A., Dasenbrock H., Holsapple J. (2025). The subdural membrane: Unraveling the pathophysiology and treatment of chronic subdural hematoma. Front. Surg..

[15] Vaibhav K., Sahu A., Prasad R.S., Deb D., Kumar D., Karimi A. (2024). Prediction of postoperative recurrence of chronic subdural hematoma using preoperative systemic immune inflammation index and eosinophils. Asian J. Neurosurg..

[16] Nakaguchi H., Tanishima T., Yoshimasu N. (2001). Factors in the natural history of chronic subdural hematomas that influence their postoperative recurrence. J. Neurosurg..

[17] Edlmann E., Giorgi-Coll S., Whitfield P.C., Carpenter K.L.H., Hutchinson P.J. (2017). Pathophysiology of chronic subdural haematoma: Inflammation, angiogenesis and implications for pharmacotherapy. J. Neuroinflamm..

